# University courses, eating problems and muscle dysmorphia: are there any associations?

**DOI:** 10.1186/s12967-014-0221-2

**Published:** 2014-08-07

**Authors:** Simona Bo, Rossana Zoccali, Valentina Ponzo, Laura Soldati, Luca De Carli, Andrea Benso, Elisabetta Fea, Alberto Rainoldi, Marilena Durazzo, Secondo Fassino, Giovanni Abbate-Daga

**Affiliations:** Department of Medical Sciences, University of Turin, c.so AM Dogliotti 14, 10126 Turin, Italy; Department of Health Sciences, University of Milan, Milan, Italy; Department of Public Health and Paediatric Science, University of Turin, P.zza Polonia 94, 10126 Turin, Italy; Department of Neuroscience, University of Turin, via Cherasco 15, 10126 Turin, Italy

**Keywords:** Eating disorders, Freshmen, Muscle dysmorphia, Orthorexia

## Abstract

**Background:**

Orthorexia and muscle dysmorphia are disorders affecting above all young adults whose prevalence and social impact are still unclear. We aimed to evaluate the prevalence of the traits of orthorexia and muscle dysmorphia among freshmen attending university courses focused on nutrition (Dietetics) and body care (Exercise and Sport Sciences). Students of Biology were considered as a control group. The prevalence of eating disorder (ED) traits were also evaluated.

**Methods:**

All participants (*n* = 440; *n* = 53 Dietetics school, *n* = 200 Exercise and Sport Sciences school, *n* = 187 the Biology school) completed the following questionnaires: ORTO-15, Muscle-Dysmorphic-Disorder-Inventory, and Eating Attitudes Test-26.

**Results:**

The prevalence of the traits of EDs, orthorexia, and muscle dysmorphia was 9.1%, 25.9%, and 5.9%, respectively. When compared to other students, those attending the Dietetics school showed a 2-fold higher risk of EDs and those from the Exercise and Sport Sciences school a 10-fold higher risk of muscle dysmorphia. The prevalence of orthorexia traits was high in all schools (35.9%, 22.5%, 26.5% in Dietetics, Biology, and Exercise and Sport Sciences schools, respectively). Overall, individuals with traits of any of these disorders were more frequently on diet or on supplement use. In a logistic regression model, attending the Dietetics school (OR = 2.71; 95% CI 1.14-6.48) was significantly associated with the ED traits, but not with the orthorexia traits (OR = 1.75; 95% CI 0.93-3.29), while attending the Exercise and Sport Sciences school was significantly associated with the muscle dysmorphia traits (OR = 5.15; 95% CI 1.44-18.4). Finally, when evaluating the relationships among the types of study programs as dependent variables and traits of these disturbances, the associations between the traits of ED (OR = 3.35; 95% CI 1.38-8.13) and matriculation at the school of Dietetics, and between the traits of muscle dysmorphia (OR = 4.32; 95% CI 1.16-16.1) and the choice of the Exercise and Sport Sciences school were confirmed.

**Conclusions:**

The choice of the university courses might be influenced by pre-existing disorders in eating behaviors, which were relatively frequent in the considered sample.

## Background

Atypical disorders in eating behaviors are increasingly described in developed countries [1]. Although the DSM-5 has renewed and extended the diagnostic categories, some eating attitudes, such as orthorexia, are still neglected and others, such as muscle dysmorphia, are not extensively considered [2]. The term orthorexia was first used in 1997 by Bratman to describe an obsession for healthy nutrition [3], characterized by an excessive and time-consuming preoccupation with healthy eating [4]. At present, orthorexia is not a formal disorder, but rather a healthy attitude: only in extreme cases, the obsessive characteristics of orthorexia become pathological, leading to a very restrictive diet with the avoidance of many foods considered to be harmful [5] and to social isolation [6]. There is no consensus either on the categorization of othorexia among mental disorders or even if it is a mental disorder, therefore it should be considered at present a controversial concept [7]. Furthermore, no comprehensive and standardized criteria for orthorexia exist [8]. The average prevalence of orthorexia, defined by Donini et al. as an attitude with obsessive-compulsive personality features plus a “fanatic” healthy eating habits, is 6.9% in the general population [4], and 35-58% in high-risk groups (healthcare professionals, such as dieticians, and artists) [8]. Among dieticians, 12.8% showed orthorexia and 34.9% some orthorectic behaviors [9]; therefore, it has been hypothesized that a disordered eating attitude might be a motivation to start a nutrition study program to cope with it [9]. Indeed, no difference for orthorexia nervosa, but only for dietary restrain were found between students attending a university course of nutrition science and the control group [10]. Finally, a great deal of controversy surrounding the notion that orthorexia is a valid nosological entity exists [11,12].

Muscle dysmorphia was first described in 1993 by Pope and coll. [13] as an obsessive-compulsive disorder, characterized by the obsession of body appearance, the fear of not being sufficiently muscled, and the compulsion to excessive physical exercise [14]. Such individuals show changes in feeding behavior, such as radical diets and dietary supplements, abuse of anabolic substances, and impairment or distress in social and physical domains [15], and in social and occupational functioning [16,17]. According to the DSM-5, the specification “with muscle dysmorphia” has been added to body dysmorphic disorder, suggesting that this is an important distinction to be done as regards this disorder. Muscle dysmorphia is more frequently diagnosed in male young adults [14,18], and bodybuilders represent a high-risk group [19]. The prevalence of muscle dysmorphia in the general population is still unknown since only few small studies are available [20]. We wonder if students from schools oriented to fitness and body care show an increased prevalence of the traits of this disorder.

Therefore, orthorexia and muscle dysmorphia seem to be attitudes with a potentially high social impact, mostly affecting young adults. We hypothesized that some students could choose a university course oriented on healthy food and healthy body, because they have a pre-existent peculiar eating behavior or pre-existing muscle dysmorphia traits. Indeed, a few studies have shown that personality traits, motivations, and lifestyle habits might correlate with the choice of the future profession [21–23]. In particular, we hypothesized that students matriculating at the school of Dietetics showed an increased prevalence of orthorexia and eating disorder (ED) traits, while those matriculating at the Exercise and Sport Sciences school showed an increased prevalence of muscle dysmorphia. The school of Biology is not specifically oriented to the body care or nutrition in Italy; we hypothesized that freshmen from this school showed a lower prevalence of all these traits.

The aim of the present study was therefore to investigate whether the prevalence of the traits of orthorexia, muscle dysmorphia, and EDs might differ among freshmen attending the schools of Dietetics and Exercise and Sport Sciences. These data were compared with those obtained from the freshmen of the school of Biology, as a control group.

## Methods

### Participants

All freshmen applying in fall 2012 to the school of Dietetics (*n* = 32), Exercise and Sport Sciences (*n* = 230), and Biology (*n* = 227) at the University of Turin were contacted. Owing to the low number of freshmen from Dietetics, those who matriculated in fall 2013 (*n* = 28) were also approached for the purposes of this study. Only the students who were about to start the different majors were considered. The students who gave their informed consent to participate were: 53/60 (88.3%), 200/230 (87.0%), and 187/227 (82.4%) from Dietetics, Exercise and Sport Sciences and Biology schools, respectively.

The study protocol was approved by the local Ethics Committee (University of Turin Bioethical Committee), and all procedures were in line with the Helsinky Declaration.

### Measures

All participants completed 4 anonymous questionnaires. The participants provided information on age, gender, ethnicity, use of drugs, dietary supplements, specific diets, and exercise level (hours/week) in the first questionnaire.

Freshmen attending Dietetics school were also asked to report their weight and height values.

The second questionnaire was the ORTO-15 test, one of the tools used to assess orthorexia [24]. This is a 15-items questionnaire, with questions rated on a 4-point scale (ranging from “never” to “always”), investigating the obsessive attitude towards the choice, preparation, and consumption of healthy foods. A lower score value (“1”) was given to the answers indicating orthorexia, while the healthier ones had a score of “4”. The sum of the points was the final score of the test; the cutoff score of <35 points showed high specificity (94.2%) and negative predictive value (91.1%) [24]. The MDDI (The Muscle Dysmorphic Disorder Inventory) was the third questionnaire; it is a 13-item tool for the diagnosis of muscle dysmorphia, containing questions on cognitions, emotions, and behaviors related to body image [25]. Items are rated on a 5-point scale, ranging from “never” to “always”; the score of the test is the sum of the scores of each item. The threshold value > 39 points showed 75% specificity and 73.7% sensitivity [26], with a Cronbach alpha coefficient = 0.85 in an Italian validation study [27].

The fourth questionnaire was the Eating Attitudes Test-26 (EAT-26), which is a widely used self-report standardized tool investigating symptoms and concerns characteristic of EDs. It is a refinement of the original EAT-40 [28]. Participants were required to judge whether “very often”, “often”, “rarely”, “sometimes” or “never” applied to each of the 26-items. A score ≥20, which is the sum of the points of each item, is considered to be predictive of the risk of EDs; the Cronbach alpha coefficient for EAT-26 is 0.86 [29]. EAT-26 is considered a highly reliable and valid instrument [30]; however, further clinical evaluations are required in order to make a correct diagnosis [31].

### Statistical analyses

All data are presented as mean ± standard deviation or percentage. Questionnaires scores and exercise levels were not normally distributed and were log-transformed, thus obtaining a normal distribution. In all the analyses the log-values were used. To improve the ease of interpretation, non-transformed values are shown.

ANOVA and χ^2^-test were used to assess the differences among groups for continuous and categorical variables, respectively. A post-hoc analysis was performed using Scheffè’s test.

A multiple logistic regression model was used to evaluate the association between the traits of ED, orthorexia and muscle dysmorphia (dependent variables), and age, gender, exercise, and the attended school. Further logistic regression analyses were performed, using two dummy coded variables in each of the models (i.e., those who matriculated at Dietetics got a score of 1 and everyone else received a 0 score, and those who majored in Exercise and Sport Sciences received a score of 1 and all others received a score of 0).

Finally, the association among the matriculation at the schools of Dietetics, Exercise and Sport Sciences, Biology (dependent variables) and the traits of ED, orthorexia, muscle dysmorphia and the respective scores, used as continuous variables was assessed by a logistic regression analysis, adjusted for age, sex, and log-exercise. One model for each variable was performed.

The p-values obtained with the likelihood ratio test with respect to the null model were given (STATISTICA software 5.1, Statsoft Italia).

## Results

The characteristics of the sample are reported in Table 1. Those attending the Dietetics school were more frequently dieting, and showed a more than 2-fold higher likelihood of EDs than other students. The students attending the Exercise and Sport Sciences school were more frequently highly exercising males on dietary supplements, and showed a 10-fold higher likelihood of having muscle dysmorphia. The prevalence of students with traits of EDs, orthorexia, and muscle dysmorphia was 9.1%, 25.9%, and 5.9%, respectively. However, the distribution of these students among schools was highly asymmetrical (Tables 1 and 2). In particular, most students with traits of muscle dysmorphia attended the Exercise and Sport Sciences school. A gender-distribution emerged amongst students with traits of EDs (mainly females) and of muscle dysmorphia (mainly males), but not for those with traits of orthorexia. Students with traits of EDs were significantly older (Table 2). Overall, individuals with traits of any of these disorders were more frequently on diet (above all students with EDs traits) or on supplement use (above all students with muscle dysmorphia traits). Among students at risk of EDs, 66.7% declared to be on a hypocaloric diet, 16.7% on a vegetarian and 16.7% on a hyperproteic regimen (p = 0.048). With respects to dieters, 45.5%, 27.3%, and 27.3% of those with traits of orthorexia and 22.2%, 33.3%, and 44.4% of those with traits of muscle dysmorphia were on hypocaloric, vegetarian, and hyperproteic diets, respectively. Furthermore, individuals with traits of any disorders were more frequently at risk of another disorder (Table 2).

**Table 1:** **Characteristics of the students grouped by school**

	**Dietetics**	**Biology**	**Exercise and Sport Sciences**	**P**
Number	53	187	200	
Males (%)	22.6	29.4##	67.5§§	<0.001
Age (years)	19.8 ± 2.7	19.7 ± 1.4	19.9 ± 1.8	0.62
Exercise (h/week)	4.4 ± 2.8	3.6 ± 2.6##	12.0 ± 6.5§§	<0.001
Score EAT-26	9.5 ± 8.1	9.3 ± 8.3	8.3 ± 7.5	0.41
Score oral control	1.7 ± 2.6	1.4 ± 2.2	1.3 ± 2.3	0.55
Score bulimia	2.1 ± 2.1	1.8 ± 2.3	1.6 ± 2.3§	0.04
Score dieting	5.8 ± 4.9	5.8 ± 4.9	5.6 ± 4.9	0.80
Traits of ED (%)	18.9*	8.6	7.0§§	0.03
Score orthorexia	37.2 ± 4.6	38.7 ± 4.8	38.6 ± 5.2	0.13
Traits of orthorexia (%)	35.9	22.5	26.5	0.14
Score muscle dysmorphia	23.3 ± 5.8	22.2 ± 9.6##	28.9 ± 7.2§§	<0.001
Traits of muscle dysmorphia (%)	1.9	1.6##	11.0§	<0.001
Supplement use (%)	5.7	7.5##	18.0§	0.002
Dieting (%)	15.1	6.4	8.0	0.12

*p < 0.05 Dietetics vs Biology.

§p < 0.05 Dietetics vs Exercise and Sport Science.

§§p < 0.01 Dietetics vs Exercise and Sport Sciences.

##p < 0.01 Biology vs Exercise and Sport Science.

**Table 2:** **Characteristics of the students according to the questionnaires**

**Traits of ED**	**Yes (** ***n*** **=** **40)**	**No (** ***n*** **=** **400)**	**P**
Males (%)	17.5	49.8	<0.001
Age (years)	20.8 ± 1.8	19.7 ± 1.7	<0.001
Exercise (h/week)	7.9 ± 3.6	7.5 ± 6.5	0.69
School: Dietetics	25.0	10.8	
Biology	40.0	42.8	
Exercise and Sport Sciences	35.0	46.5	0.03
Supplement use (%)	20.0	11.3	0.11
Dieting (%)	45.0	4.5	<0.001
Traits of orthorexia (%)	70.0	21.5	<0.001
Traits of muscle dysmorphia (%)	15.0	5.0	0.01
**Traits of orthorexia**	**Yes (** ***n*** **=** **114)**	**No (** ***n*** **=** **326)**	**P**
Males (%)	40.4	49.1	0.11
Age (years)	20.1 ± 1.5	19.7 ± 1.8	0.10
Exercise (h/week)	7.9 ± 5.7	7.4 ± 6.5	0.40
School: Dietetics	16.7	10.4	
Biology	36.8	44.5	
Exercise and Sport Sciences	46.5	45.1	0.14
Supplement use (%)	19.3	9.5	0.006
Dieting (%)	19.3	4.3	<0.001
Traits of ED (%)	24.6	3.7	<0.001
Traits of muscle dysmorphia (%)	9.7	4.6	0.05
**Traits of muscle dysmorphia**	**Yes (** ***n*** **=** **26)**	**No (** ***n*** **=** **414)**	**P**
Males (%)	73.1	45.2	0.006
Age (years)	20.2 ± 1.8	19.8 ± 1.7	0.31
Exercise (h/week)	11.9 ± 7.7	7.3 ± 6.1	<0.001
School: Dietetics	3.8	12.6	
Biology	11.5	44.4	
Exercise and Sport Sciences	84.6	43.0	<0.001
Supplement use (%)	61.5	8.9	<0.001
Dieting (%)	30.8	6.8	<0.001
Traits of ED (%)	23.1	8.2	0.01
Traits of othorexia (%)	42.3	24.9	0.05

Weight and height of the Dietetics school students (*n* = 53) were self-reported. Mean weight and BMI of students with traits of EDs (*n* = 10) were 47.0 ± 3.9 kg and 16.9 ± 1.6 kg/m^2^ while the corresponding values of students without traits (*n* = 43) were 64.7 ± 12.7 kg and 22.4 ± 3.3 kg/m^2^ (gender- and age-adjusted p values were < 0.001 for both weight and BMI).

The association between disorders and the school choice was evaluated in a logistic regression model, after adjusting for age, gender, and exercise level (Table 3). Female gender (OR = 0.15; 95% CI 0.06-0.39 for male gender), age (OR = 1.38; 95% CI 1.15-1.66), and attending the Dietetics school (OR = 2.71; 95% CI 1.14-6.48) resulted significantly associated with the likelihood of having EDs (Table 3), but not with the likelihood of orthorexia. Both male gender and exercise level were significantly associated with the likelihood of having muscle dysmorphia at univariate analysis; these associations disappeared in a multivariate regression analysis, after introducing into the model the matriculation at the Exercise and Sport Sciences school which resulted the only variable to be significantly associated with the likelihood of muscle dysmorphia (OR = 5.15; 95% CI 1.44-18.4) (Table 3).

**Table 3:** **Conditions associated with traits of EDs, orthorexia and muscle dysmorphia in a multiple logistic regression model**

**Traits of eating disorders**	**β**	**SE β**	**Wald**	**P**	**OR**	**95%CI**	P**
Age	0.32	0.09	11.8	0.001	1.38	1.15-1.66	
Males	−1.87	0.47	15.7	<0.001	0.15	0.06-0.39	
Exercise*	0.69	0.39	3.13	0.08	1.99	0.93-4.31	
School of Dietetics	1.00	0.44	5.09	0.02	2.71	1.14-6.48	<0.001
**Traits of orthorexia**							
Age	0.11	0.06	3.09	0.08	1.11	0.99-1.26	
Males	−0.43	0.23	3.39	0.07	0.65	0.41-1.03	
Exercise*	0.34	0.25	1.90	0.17	1.41	0.86-2.29	
School of Dietetics	0.56	0.32	3.01	0.08	1.75	0.93-3.29	0.04
**Traits of muscle dysmorphia**							
Age	0.07	0.10	0.53	0.47	1.08	0.88-1.31	
Males	0.59	0.48	1.48	0.22	1.80	0.70-4.63	
Exercise*	0.20	0.48	0.19	0.67	1.23	0.48-3.13	
School of Exercise and Sport Sciences	1.64	0.65	6.39	0.01	5.15	1.44-18.4	<0.001

*Higher quartile of exercise *vs* the lower quartiles; **p-values of the likelihood ratio test.

By using both the school of Dietetics and the school of Exercise and Sport Sciences as two dummy variables in the logistic regression analyses, matriculating at the Dietetics school was significantly associated with traits of EDs (OR = 2.54; 95% CI 1.02-6.40; p = 0.04) and matriculating at the school of Exercise and Sport Sciences was associated with traits of muscle dysmorphia (OR = 4.62; 95% CI 1.15-18.6; p = 0.03).

Finally, when evaluating the relationships among the types of study programs as dependent variables (Table 4) and traits or scores of the three disturbances, the associations between the traits of ED (OR = 3.35; 95% CI 1.38-8.13) and the bulimia score (OR = 1.10; 95% CI 1.02-1.22) with matriculation at the school of Dietetics were confirmed, while the associations between orthorexia traits (OR = 1.87; 95% CI 1.00-3.52) and score (OR = 0.94; 95% CI 0.88-1.00) and this school resulted borderline (Table 4). Furthermore, the associations between muscle dysmorphia traits (OR = 4.32; 95% CI 1.16-16.1) and score (OR = 1.07; 95% CI 1.02-1.13) and the choice of the Exercise and Sport Sciences school were confirmed too. An inverse association between the muscle dysmorphia score and the choice of the Biology school was found.

**Table 4:** Association between the type of study program and the traits of EDs, orthorexia and muscle dysmorphia in a multiple logistic regression model*

**School of Dietetics**	**Β**	**SE β**	**Wald**	**P**	**OR**	**95%CI**	p**
Traits of ED	1.21	0.45	7.25	0.007	3.35	1.38-8.13	<0.001
Score EAT-26	0.01	0.02	0.35	0.56	1.01	0.97-1.05	0.009
Score oral control	0.04	0.06	0.53	0.47	1.04	0.93-1.18	0.009
Score bulimia	0.10	0.05	3.75	0.04	1.10	1.02-1.22	0.005
Score dieting	0.01	0.03	0.20	0.66	1.01	0.95-1.08	0.01
Traits of orthorexia	0.62	0.32	3.75	0.05	1.87	1.00-3.52	0.002
Score orthorexia	−0.06	0.03	4.32	0.05	0.94	0.88-1.00	0.002
Traits of muscle dysmorphia	−0.87	1.04	0.70	0.40	0.42	0.05-3.26	0.007
Score muscle dysmorphia	−0.01	0.02	0.35	0.55	0.99	0.95-1.03	0.009
**School of Exercise and Sport Sciences**							
Traits of ED	−0.48	0.35	1.91	0.17	0.62	0.31-1.22	<0.001
Score EAT-26	−0.02	0.01	1.86	0.17	0.98	0.96-1.01	<0.001
Score oral control	−0.03	0.05	0.29	0.59	0.97	0.88-1.08	<0.001
Score bulimia	−0.08	0.05	3.43	0.06	0.92	0.84-1.01	<0.001
Score dieting	−0.03	0.02	1.95	0.16	0.97	0.92-1.01	<0.001
Traits of orthorexia	−0.16	0.30	0.29	0.59	0.85	0.47-1.53	<0.001
Score orthorexia	0.01	0.03	0.32	0.57	1.01	0.96-1.07	<0.001
Traits of muscle dysmorphia	1.46	0.67	4.79	0.03	4.32	1.16-16.1	<0.001
Score muscle dysmorphia	0.10	0.01	12.8	<0.001	1.07	1.02-1.13	<0.001
**School of Biology**							
Traits of ED	0.26	0.40	0.44	0.51	1.30	0.60-2.84	<0.001
Score EAT-26	0.01	0.01	0.89	0.35	1.01	0.98-1.04	<0.001
Score oral control	0.01	0.05	0.03	0.86	1.01	0.92-1.11	<0.001
Score bulimia	0.04	0.04	1.10	0.29	1.05	0.96-1.14	<0.001
Score dieting	0.04	0.02	2.46	0.12	1.04	0.99-1.08	<0.001
Traits of orthorexia	−0.19	0.27	0.51	0.48	0.82	0.48-1.40	<0.001
Score orthorexia	0.02	0.02	0.43	0.51	1.02	0.97-1.07	<0.001
Traits of muscle dysmorphia	−1.08	0.68	2.58	0.11	0.34	0.09-1.28	<0.001
Score muscle dysmorphia	−0.05	0.02	7.97	0.005	0.95	0.92-0.98	<0.001

*Multiple logistic regression model, adjusted for age, sex, log-exercise (one model for each row); **p-values of the likelihood ratio test.

## Discussion

The prevalence of EDs traits was high among female freshmen attending the school of Dietetics, while that of muscle dysmorphia traits was higher among males attending the Exercise and Sport Sciences school, as expected. Contrarily to our hypothesis, orthorexia was a condition for which about one fourth of the students across different schools were at risk, without a clear gender distribution.

Only few studies are available on this topic. A European survey found that 12.8% of dieticians showed symptoms of orthorexia and reported a previous or current ED, such as anorexia nervosa, bulimia nervosa or binge eating disorder [9]. Accordingly, we found that about one fifth of students attending the school of Dietetics showed traits of EDs. Our results were higher than those reported in another Italian study where a 5.5% prevalence was found in high-school students in Turin [32]. Therefore, female students attending the Dietetics school could be at greater risk for EDs than younger females [9]. Indeed, our prevalence of individuals with traits of ED attending the Exercise and Sport Sciences and Biology schools was similar to that shown by the aforementioned Italian study [32]. Furthermore, adults might be more likely to report their eating problems than adolescents.

Another German cross-sectional study found that students attending higher semesters of Universities in nutrition sciences had lower scores on dietary restraint than younger students, and that they adopted slightly more healthy food choices [10]. Similar results were found in an US study among students enrolled in Dietetics, which found that junior and senior majors have more positive eating patterns than freshmen as a result of their increased exposure to nutritional information [9]. Our results confirmed the hypothesis that individuals with pre-existing pathological eating behaviours may be inclined to attend the school of Dietetics.

In our study, we found that students of Dietetics with traits of EDs were older than those without these traits. The age range of EDs onset is 18–20 years, but a later onset has been described [33]. The students of the Exercise and Sport Sciences school showed a more than 5-fold increased adjusted risk of muscle dysmorphia. Most literature on this topic focused on bodybuilders and weightlifters who represent the groups at greatest risk of muscle dysmorphia [34]. An early research proposed that up to 10% of bodybuilders may be afflicted by this disorder [19], but it is difficult to estimate the prevalence of muscle dysmorphia among the general population and only few studies included mixed-gender samples [20]. It is widely accepted that this condition is substantially a male disturbance; however, it can also affect women [35], and other studies did not find significant differences between genders [36]. We found 7/26 (27%) female students with these traits and this finding seems worth further studying with larger samples to analyze whether affected girls show any specific behavior and different psychopathological patterns. For example, all females with traits of muscle dysmorphia in our sample were on diet, while the corresponding males reported to be on ergogenic supplement use. Accordingly, individuals with muscle dysmorphia are described to abuse of anabolic-androgenic steroids and supplements, such as protein, amino acids and vitamins [17,37]. It is possible that females at risk of muscle dysmorphia show some psychological traits similar to those of females at risk for EDs, while males at risk are characterized by obsessive-compulsive traits [34]. Indeed, different clinical forms of muscle dysmorphia and the frequent co-presence of an ED in dysmorphic females have been described by the DSM-5 [2]. Further studies are needed to clarify these psychopathological issues. Intriguingly, we found an inverse association between the score of muscle dysmorphia and the school of Biology, thus confirming the relationship between some disturbances and the selection or exclusion of the schools.

Orthorexia was the most frequent disturbance in our cohort. The prevalence we obtained (about 25%) was higher than that found in the general population (about 7%) [25], but in line with previous results deriving from high-risk groups [8]. Studies on specific subgroups reported prevalence greater than ours: 45% among medical doctors [5], and 56% among performance artists (opera singers, ballet dancers, and musicians) [38]. No significant difference by gender emerged among our students with orthorexia traits, contrarily to literature where either a male [25,39] or a female [40] predominance were found. Contrarily to our hypothesis, the prevalence of the orthorexia traits was high in the control group, namely the students attending the school of Biology. The boundaries between healthy and pathological might be more difficult to be defined for the orthorectic attitude and it may represent a consequence of the greater attention to the nutritional education in the youths occurring in recent years, given the uniform distribution of this attitude among schools. Finally, at present, this entity is far from being well defined and characterized, and more specific criteria or validated tools for its diagnosis are needed.

EDs, muscle dysmorphia, and orthorexia are different conditions; in accordance with the DSM-5 classification muscle dysmorphia has been classified as an obsessive-compulsive disorder (i.e. not an ED), while at present orthorexia has not been included at all. However, in our cohort these attitudes were frequently associated, particularly orthorexia and EDs. Indeed, even if these disorders are different, the latter two are both characterized by a common problem: a controlled feeding behavior [41].

### Limitations

This study suffers from several limitations. It is an observational study and the possibility of residual confounding cannot be excluded. Anthropometric variables were not measured; in the subgroup of students from the Dietetics school, however, the referred values of weight and the calculated BMI corresponded to the EDs traits. No psychiatric evaluation was performed, data were obtained by self-report questionnaires and not all the used tools have received an appropriate validation. Indeed, our results were consistent with clinical and literature data. For example, the findings that two thirds of our students with ED traits declared to be on a hypocaloric diet and most of those with muscle dysmorphia traits were on hyperproteic diets seem to confirm that diet behaviors are more frequently associated with restrictive or selective feedings [36,42]. Dieting is a well known risk factor for EDs [43], but it is important not to pathologize it in the absence of other risk factors, because only 1% of the dieting adolescents will develop an ED [43,44].

We cannot exclude that a cohort effect due to the assessment of the freshmen from the Dietetic school in the year 2012 and 2013 might exist and impact on the associations found. Furthermore, caution should be exercised in interpreting the finding of this study, owing to the low number of subjects studied. Larger studies may be needed to generate more precise findings; data relative to schools unrelated to the study of the living organism might be interesting too.

Finally, our results may be culture-specific and might not be applicable to other cultures or ethnic groups.

## Conclusion

The choice of the University courses might be influenced by pre-existing disorders in eating behaviors. These results are worthy to be confirmed in larger and longitudinal studies, because of their health implications and of the preventive measures potentially required.

## Abbreviations

BMI: Body mass index
DSM-5: Diagnostic and statistical manual of mental disorders, fifth edition
EAT-26: Eating Attitude Test-26
EAT-40: Eating Attitude Test-40
ED: Eating disorders
MDDI: Muscle dysmorphic disorder inventory
